# Difficulties in diagnosing tuberculosis in pregnancy

**DOI:** 10.4103/0256-4947.51804

**Published:** 2009

**Authors:** Muneerah Albugami, Abdulaziz Tashkandi, Abdulaziz AlRashed

**Affiliations:** a Section of Internal Medicine, King Faisal, Specialist Hospital and Research Centre, Riyadh, Saudi Arabia; b Department of Medicine, King Faisal, Specialist Hospital and Research Centre, Riyadh, Saudi Arabia

**To the Editor:**

Diagnosing tuberculosis (TB) in pregnancy can be difficult because of the vague, non-specific nature of the symptoms. Fatigue, shortness of breath, sweating and tired, all characteristic of TB, can also be due to pregnancy. Most physicians are reluctant to order a chest x-ray for fear of harming the fetus. This case illustrates the problems in early recognition and management of TB in pregnancy.

An 18-week pregnant woman who had a lowbgrade fever for 3 months underwent an extensive and unnecessary work up for pyrexia of unknown etiology. All tests were normal including a bone marrow biopsy. Due to concerns over harming the fetus, a chest x-ray was not performed. MRI of the chest and abdomen showed a miliary pattern. The diagnosis was tuberculosis (TB). A sputum culture was positive for acid-fast bacilli and sensitive to first-line anti-TB therapy. A chest x-ray requested 2 weeks after admission showed the miliary shadow. A PPD skin test was negative. The patient received anti-TB therapy for 9 months without complications. She had a baby girl who was healthy.

Treatment of TB in pregnancy is multidisciplinary, requiring input from all physicians involved in the care of the mother and fetus. Between 20% and 67% of pregnant patients presenting with pulmonary TB are unaware of their disease and have no significant symptoms.^1^ Available information suggests that pregnancy has little, if any, effect on the clinical course of TB.^2^ Screening for TB in pregnancy is not required because of the reluctance to do a chest x-ray on a pregnant women. A review of TB diagnoses in Rhode Island, USA from 1987 to 1991 found that pregnant women with TB were most likely to be identified through routine screening and to be asymptomatic.^1^

If the chest x-ray is necessary, suitable shielding will limit fetal radiation exposure to less than 0.3 mrads and should not harm the fetus.^3^ The perception of teratogenic risk is higher than the actual risk.^4^ MRI uses electromagnetic radio waves, rather than ionizing radiation, to generate detailed computer images. There are no reported harmful effects from MRI on the pregnant woman or fetus.^5^ However, MRI is not recommended in the first trimester since safety information during organogenesis is limited. A TB skin test using purified protein derivative (PPD) is safe and accurate during pregnancy and is recommended for women who have TB symptoms or are at high risk for TB. It is as reliable as in non-pregnant women.^6^,^7^ No evidence suggests that TB affects or complicates either the course of pregnancy or delivery; women with and without TB have similar rates of normal, spontaneous deliveries.^9^ Combination anti-TB therapy in pregnancy is safe and effective. Most experts consider the drugs of choice to be rifampin, combined with isoniazid and ethambutol.^10^ All these drugs cross the placenta and reach low concentrations in human fetal fluids and tissues, but there are no reports linking the use of these drugs with congenital anomalies. The following anti-TB drugs are contraindicated in pregnant women: streptomycin, kanamycin, amikacin, capreomycin and the fluoroquinolones. Women on second-line agents for multi-drug resistant TB need to be assessed on an individual basis regarding their risks. Women who become pregnant on anti-TB therapy can be reassured that there is no increased risk to their baby, and they should complete their treatment course.^8^–^10^

Breastfeeding should not be discouraged for women being treated with the first-line anti-TB drugs because the concentrations of these drugs in breast milk are too small to produce toxicity in the nursing newborn.^8^

Congenital TB in neonates has morbidity and mortality approaching 50%.^11^ About 200 cases have been reported in the English literature.^12^,^13^ The child can be infected in the uterus if the mother has had miliary TB, TB of the placenta or uterus, or has advanced HIV.
